# PIGMENTED CONTACT CHEILITIS TO PARAPHENYLENEDIAMINE

**DOI:** 10.4103/0019-5154.60370

**Published:** 2010

**Authors:** Vandana Mehta, Sudhir Nayak, C Balachandran

**Affiliations:** *From the Department of Skin and STD, Kasturba Hospital, Manipal - 576 104, Karnataka, India. E-mail: vandanamht@yahoo.com*

Sir,

Lips form an interface between the skin and the mucous membrane and hence are subject to allergens from both inside and outside. With emphasis being now on good looks, the use of cosmetics and dyes is quite rampant. Lip cosmetics are the most common cause of allergic contact cheilitis. It has been said that the flavouring agents and preservatives used in lipsticks are the most common allergens rather than the dyes. Paraphenylene diamine (PPD) is a coal-tar derivative that is widely used as a permanent hair dye. It is one of the causative allergen in cases of pigmented cosmetic dermatitis occurring on the face in the Indian population. We herewith report a case of pigmented contact cheilitis to PPD in a male who had no evidence of facial pigmentation.

**Figure 1:**
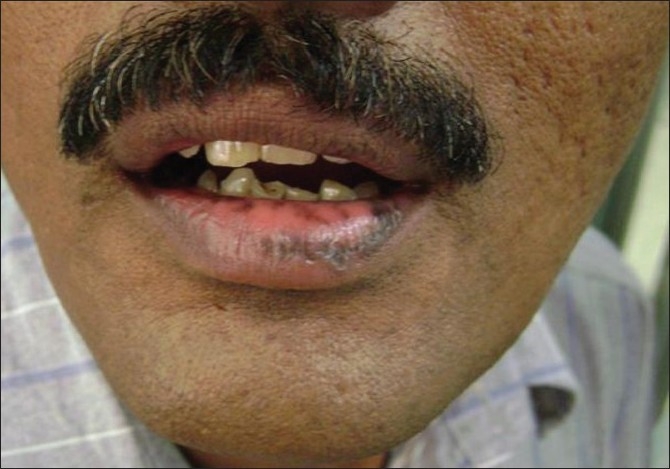
Hyperpigmented macules with mild scaling on the lower lip

A 47-year-old male presented with complaints of hyperpigmentation over his lips for the past few months. There was neither history of itch nor any cosmetic use on the lips or body elsewhere; however, he gave a history of use of hair dyes for the scalp and moustache hair for many years. His past medical history was unremarkable. The cutaneous examination of the lower lip and the mucosal aspect revealed hyperpigmented macules with scaling. [Figure 1] A patch test was performed with Indian standard series, which showed strong positive reactions to PPD and fragrance mix at 48 and 72 h. Taking the history and patch test findings into consideration, a final diagnosis of contact cheilitis to PPD was made. Treatment was commenced with topical steroids and hydroquinone with further advice to avoid the use of PPD containing hair dyes.

PPD is a very strong sensitizer and a common contact allergen in hair dyes. It is used to speed up the process of hair dyeing as it gives a dark brown to black color to the hair. PPD is known to induce allergy *per se* or be a marker of allergy to other diaminobenezenes.[1] PPD-induced acute irritant dermatitis is well known, but uncommon presentations such as lichen planus like, erythema multiforme like, and pigmentary changes have also been described with its use. Pasricha *et al*. described positive patch test with PPD in 42% cases (61/144) and 40% (57/144) with hair dyes.[2] Dogra *et al*. showed 35% sensitivity with PPD in hair dyes.[3] In an another study done by Dogra *et al*., 45% (9/20) patients of hair dye dermatitis developed reaction to PPD.[4] PPD may cross react with various other compounds such as azo dyes, aniline dyes, local anaesthetics, sunscreens containing PABA, sulphonamides, hydroquinones, parahydroxybenzoic acid esters, phenyl hydrazine etc.[5], which could be a reason why PPD is a very common allergen.

Pigmented contact dermatitis is a noneczematous variant of contact dermatitis, characterized clinically by hyperpigmentation, with subtle or no signs of dermatitis. There is paucity of data available on the occurrence of pigmented contact dermatitis in Indian population and the most common allergen implicated is red kumkum.[6] Our patient did not have any facial hypermelanosis but only lip pigmentation. He had applied hair dye not only to the scalp but also to the moustache hair and presented to us with contact cheilitis. Many patients show positivity to their personal products and it is prudent for the clinician to elicit a history of these, thus making history taking an important tool in the diagnosis. History of usage of hair dye only in the moustache area might be skipped unless specifically asked for.

To conclude, the avoidance of PPD in those allergic to it is always advised, but more essential is that the hair dye manufactures should either avoid PPD as a component of hair dyes or make a bold mention of it in the package so as to avoid the occurrence of contact dermatitis.
